# Development of retinal structure in perinatally HIV-infected children and adolescents: A longitudinal and cross-sectional assessment

**DOI:** 10.1371/journal.pone.0282284

**Published:** 2023-03-02

**Authors:** Jason G. van Genderen, Charissa R. Verkade, Malon Van den Hof, Nazli Demirkaya, Anouk G. M. Schrantee, Frank D. Verbraak, Dasja Pajkrt

**Affiliations:** 1 Department of Pediatric Infectious Diseases, Emma Children’s Hospital, Amsterdam UMC, Location Academic Medical Center, Amsterdam, the Netherlands; 2 Department of Ophthalmology, Amsterdam UMC, Location Academic Medical Center, Amsterdam, the Netherlands; 3 Department of Radiology and Nuclear Medicine, Location Academic Medical Center, Amsterdam University Medical Centers, University of Amsterdam, Amsterdam, the Netherlands; UPMC and Pittsburgh School of Medicine, UNITED STATES

## Abstract

In perinatally HIV-infected (PHIV) children, cross-sectional studies reported on subtle structural retinal differences and found associations between the retina and structural brain changes. Our objective is to investigate whether neuroretinal development in PHIV children is similar to the development in healthy matched controls and to explore associations with the brain structure. We measured RT using optical coherence tomography (OCT) on two occasions in 21 PHIV children or adolescents and 23 matched controls–all with good visual acuity–with a mean interval of 4.6 years (SD 0.3). We also included 22 participants (11 PHIV children and 11 controls) together with the follow-up group for a cross-sectional assessment using a different OCT device. Magnetic resonance imaging (MRI) was used to assess the white matter microstructure. We used linear (mixed) models to assess changes in RT and its determinants (over time), adjusting for age and sex. The development of the retina was similar between the PHIV adolescents and controls. In our cohort, we found that changes in the peripapillary RNFL was significantly associated with changes in WM microstructural makers: fractional anisotropy (coefficient = 0.030, p = 0.022) and radial diffusivity (coefficient = -0.568, p = 0.025). We found comparable RT between groups. A thinner pRNFL was associated with lower WM volume (coefficient = 0.117, p = 0.030). PHIV children or adolescents appear to have a similar development of the retinal structure. In our cohort, the associations between RT and MRI biomarkers underscore the relation between retina and brain.

## Introduction

Despite effective combination antiretroviral therapy (cART), subtle ocular abnormalities are still reported in adults with behaviourally acquired human immunodeficiency virus (HIV)-infection and perinatally HIV-infected (PHIV) children [1–5]. In HIV-infected adults, some studies reported a thinner macula, while other studies found a thicker macula or no difference as compared to healthy controls [1,6–9].

Previous studies investigating neuroretinal changes in PHIV children are scarce and cross-sectional. We previously showed a significantly thinner fovea in PHIV children on long term cART treatment, compared to matched controls [5]. Two earlier studies reported a significant thinner pRNFL and a thicker fovea in PHIV children treated with cART compared to controls [3,4].

We previously reported an association between decreased retinal thickness (RT) and reduced white matter (WM) microstructure in PHIV children and a positive association between RT and grey matter (GM) and WM volume in healthy controls [10].

The pathophysiological mechanisms underlying the effects of HIV on the retina are incompletely understood, though it is hypothesized that it is part of a neuroretinal disorder which includes microvascular pathology and cART neurotoxicity [11–13]. It may also be associated with cerebral pathology caused by ongoing immune activation, in utero exposure or irreversible damage caused by the HIV infection before cART treatment was initiated [14–16].

To date, there are no longitudinal data on neuroretinal development in PHIV children or adolescents. As PHIV children are growing into adulthood due to effective treatment, it is important to investigate possible HIV-associated damage which might lead to visual complications. Several studies reported associations between thinning of the retina and function like contrast sensitivity and colour vision in HIV-infected adults [17,18]. In PHIV children this association was not found [5]. To gain further insight in the development of neuroretinal alterations in PHIV adolescents and children, we assessed their RT longitudinally and cross-sectionally. We also explored possible associations with HIV and cART-related characteristics and the white matter microstructure as previous studies reported significant associations with the RT [5,10].

## Methods

We included participants from the neurologic, cognitive and visual performance in PHIV children (NOVICE) study. This cohort study assessed neurological, cognitive and visual performance in PHIV children and adolescents compared to healthy controls matched for age, sex, socio-economic status and ethnic background. The first assessment took place between December 2012 and January 2014 as previously published [19]. Between February 2017 and July 2018 all PHIV children and adolescents, aged 8 years or older, visiting the outpatient clinic of the Academic Medical Centre, were approached again for participating in a second assessment and those who provided consent were included. This study consisted of two substudies. For the longitudinal substudy we used data from participants who were assessed twice. For the cross-sectional substudy, we included all participants from the second assessment, including participants who participated for the first time. The matched control group was selected through parental evenings at schools, churches and sport clubs [20]. We used the following exclusion criteria for both substudies: children with a history of chronic non-HIV-associated neurological diseases like epilepsy, intracerebral neoplasms and psychiatric disorders. Exclusion criteria for the ophthalmic examination were high refractive errors (spherical equivalent > +5.5 or <-8.5 dioptres), visual acuity above a 0.1 logarithm of the Minimum Angle of Resolution (logMAR), an intraocular pressure higher than 21 mmHg and a history of ocular surgeries and all inflammatory ocular diseases, specifically cytomegalovirus (CMV) retinitis. A full list of exclusion criteria was published in detail previously [20]. The study protocol followed the guidelines provided by the ethics committee of the Amsterdam Medical Centre and adhered to the tenets of the Declaration of Helsinki. Written informed consent was obtained from all participants older than 12 years and from parents of participants younger than 18 years of age. The NOVICE study was registered with the Dutch Trial Register (Netherlands Trial Register) as NTR4074.

We used data on demographics either through previous inclusion or through questionnaires for newly included participants. These characteristics included sex, age, ethnic background and adoption status [19]. We assessed the available HIV RNA viral loads (VL) and CD4+ T-cell of the PHIV children or adolescents. The Dutch HIV Monitoring Foundation database provided data on historical HIV-and cART-related characteristics [21]. We confirmed a negative HIV status for controls.

**Fig 1:**
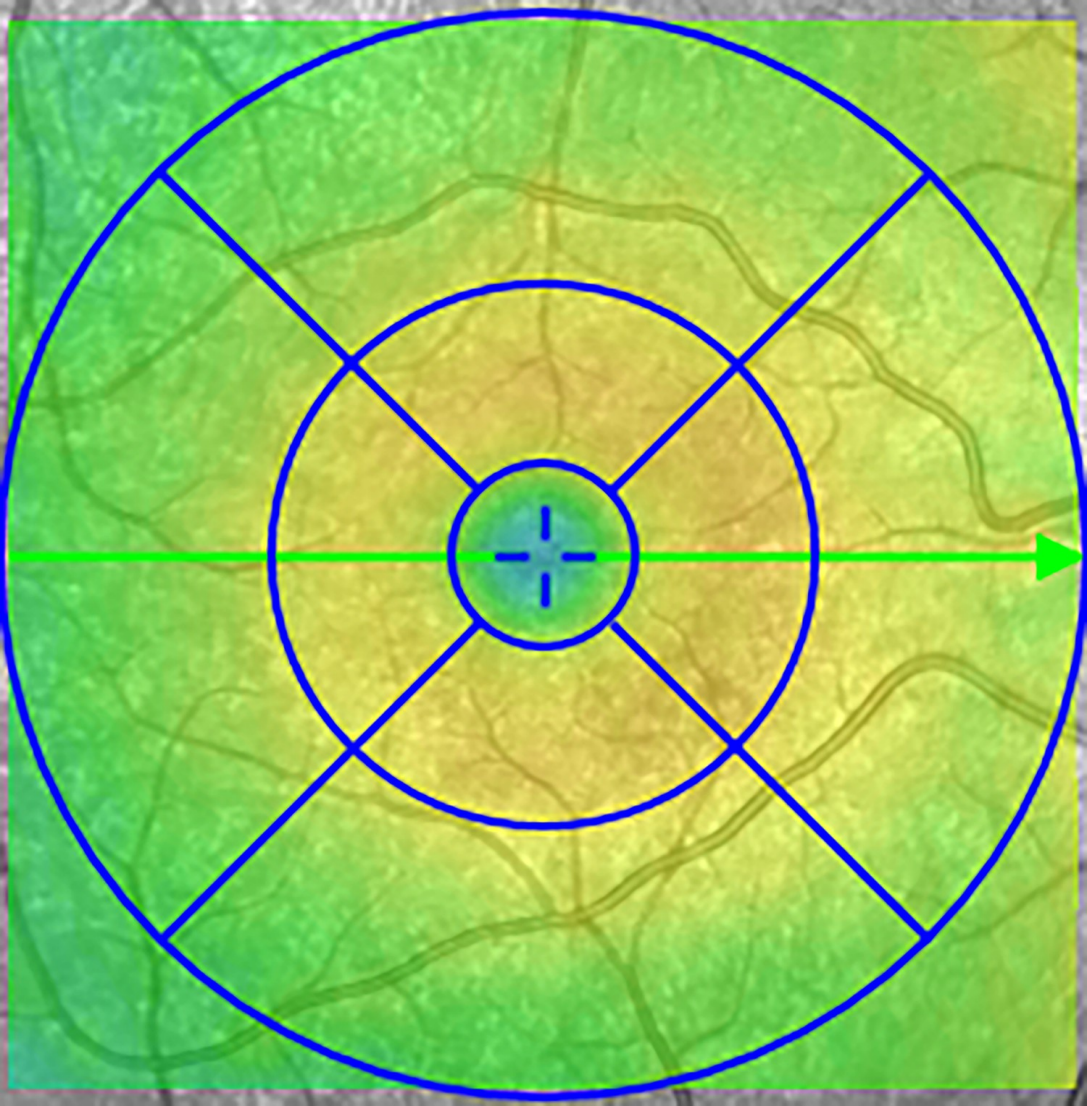
ETDRS grid. ETDRS grid: Fovea, in the middle of the circle with a diameter of 1mm; pericentral ring, area around the fovea with diameter of 3mm; peripheral ring, outer ring of the circle with a diameter of 6mm. Abbreviation: ETDRS = Early Treatment Diabetic Retinopathy Study.

**Fig 2:**
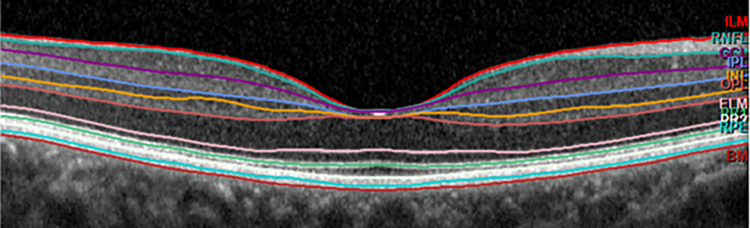
Macular Heidelberg spectralis scan with the individual retina layers. From top to bottom: Choroid; RPE, retina pigment epithelium; PR, photoreceptors; OPL, outer plexiform layer; INL, inner nuclear layer; IPL, inner plexiform layer; GCL, ganglion cell layer; RNFL, retina nerve fibre layer; ILM, inner limiting membrane.

To account for methodological sound comparison in the longitudinal study we used similar OCT equipment to measure RT, namely the spectral-domain-OCT (SD-OCT) (Topcon 3D OCT-1000; Topcon, Inc., Paramus, NJ, USA). The SD-OCT calculates the thickness of the total retina layers for each region of the ETDRS grid defined regions (Fig 1). These regions are centred around the maculae with the fovea in the middle with a pericentral and peripheral ring. The RT from both visits was reassessed by the same investigator (CV). We used the non-invasive Heidelberg Spectralis OCT (Heidelberg Engineering, Heidelberg, Germany) for the cross-sectional study. The Heidelberg Spectralis incorporates software (version 1.9.10.0) calculating the thickness of individual retinal layers for each region of the ETDRS grid defined regions. The Heidelberg Spectralis OCT segments seven individual layers and three combined layers (Fig 2). In addition, the Heidelberg Spectralis OCT measured the thickness of the pRNFL, the RNFL around the optic nerve. The pRNFL measurement was repeated in case there was doubt about the quality of the scans and in case of additional good quality scans the average outcome was calculated. We included only the measurements with a quality factor (QF) of 20 decibel or higher.

Advanced MRI brain scans were performed using a 3.0 Tesla MRI scanner (Intera, Philips Healthcare, Best, The Netherlands). In the longitudinal substudy, participants were scanned at both study visits. We used WM integrity (indicative of WM microstructural damage), white matter hyperintensities (WMH) and brain volumes to evaluate potential associations with changes in OCT outcomes, due to previously found cross-sectional associations [10]. Diffusion tensor imaging (DTI) was used to obtain fractional anisotropy (FA), mean diffusivity (MD), axial diffusivity (AD) and radial diffusivity (RD) representing WM integrity. We also used GM, WM and WMH as variables. Further details of the acquisition and processing of MRI data–including MRI parameters–were previously published in detail [19]. Specifically, we used ITK-SNAP version 3.4.0 to segment WMH [22].

### Statistical analysis

For the statistical analysis we used R version 4.1.0 [23]. We compared the demographic characteristics between the two groups using the unpaired t-test for normally distributed continuous data, the Mann-Whitney *U* test for non-normally distributed continuous data and the Fisher’s exact test for categorical data. We assessed selective drop-out by comparing OCT outcomes at first assessment between PHIV children or adolescents and controls who did or did not participate at follow-up. For the analyses of all the retinal measurements, we used the mean measurement of both eyes. When a scan was missing or contained an error, we excluded this scan and we used the measurement of one eye. To determine RT development, we evaluated the group-by-time interaction of OCT parameters over time using linear mixed models. For the cross-sectional study, we compared the outcomes between PHIV children or adolescents and healthy controls using linear regression models. We explored potential associations between the differences over time and cross-sectionally in OCT and MRI parameters in the PHIV children and controls together. We adjusted all models for age and sex. We used the following ophthalmologic parameters for the associations: total RT of the fovea, the pericentral area and the peripheral area. We also included the RNFL, ganglion cell layer (GCL) and pRNFL for the association analyses as these contribute to the optic nerve and are directly connected to the brain. We used the following MRI parameters: FA, AD, MD and RD, GM and WM volume and WMH volume. The GM and WM volume and the FA was multiplied by 10^2^ and the other DTI parameters by 10^6^ for better interpretation of coefficients. We logarithmically transformed the WMH volume to approach a normal distribution. We considered a *p* value < 0.05 as significant. Our association analyses are considered explorative, hence we did not correct for multiple analyses. We calculated the effect sizes for the cross-sectional and the longitudinal study using Cohen’s *d*.

## Results

### Demographic characteristics of the longitudinal substudy

For the longitudinal substudy, 21 of 34 (62%) PHIV children and 23 of 37 (62%) healthy matched controls consented for a second assessment with a mean follow-up of 4.6 years (SD 0.3) (Table 1A). Reasons not to participate for the follow-up study were unwillingness (PHIV 9; controls 9), inability to contact (PHIV 0; controls 5) or relocation (PHIV 2; controls 0). There was no significant difference in the participants’ demographic characteristics between those who participated in the longitudinal substudy and those who did not.

**Table 1:** a. Participants’ Characteristics at 2^nd^ enrollment of the longitudinal substudy. b. Participants’ characteristics of the cross-sectional substudy.

Demographic characteristics	n	PHIV adolescents	n	Healthy controls	*p*
Consenting to second OCT	34	21 (62%)	37	23 (62%)	
OCT of good quality 1^st^ visit*	42	40 (95%)	46	42 (91%)	
OCT of good quality 2^nd^ visit*	42	42 (100%)	46	42 (91%)	
Interval between OCT scans (y)		4.6 (0.3)		4.6 (0.3)	
Age first visit (y)	21	13.4 (10.9–15.6)	23	12.1 (11.1–15.2)	.605^C^
Age second visit (y)	21	17.5 (15.5–20.7)	23	16.4 (15.8–19.5)	.538^C^
Male sex	21	12 (57%)	23	9 (39%)	.372^B^
Ethnic background	21		23		.740^B^
Black		16 (76%)		16 (70%)	
Other		5 (24%)		7 (30%)	
Adopted	21	7 (33%)	23	0 (0%)	**.003** ^**B**^
Age at HIV diagnosis (y)	21	1.7 (0.8–4.2)			
CDC categoryƚ	21				
N/A		8 (38%)			
B		8 (38%)			
C		5 (24%)			
Peak HIV VL log copies/mL	18	5.5 (5.0–5.8)			
Nadir CD4+ *Z* score	19	-0.82 (0.61)			
HIV viral load at eye exam	21				
Undetectable at 1^st^ eye exam		19 (90%)			
Undetectable at 2^nd^ eye exam		17 (81%)			
Undetectable during follow-up		15 (71%)			
Age at cART initiation (y)	18	2.5 (1.2–6.0)			
Current cART use	21	20 (95%)			
Duration cART use (y)	18	14.9 (9.5–19.6)			
**Demographic Characteristics**	**n**	**PHIV children**	**n**	**Healthy controls**	***p***
Male	32	16 (50%)	34	15 (44%)	.817^B^
Age	32	15.4 (11.2–19.2)	34	15.3 (10.9–17.6)	.408^C^
Adopted	32	17 (53%)	34	12 (35%)	.215^B^
Ethnic background	32		34		.099^B^
Black		27 (84%)		25 (74%)	
Other		5 (15%)		9 (27%)	
OCT of good quality per eye	64	62 (97%)	68	66 (97%)	
Age at HIV diagnosis (y)	32	2.1 (0.8–4.1)	** **		
CDC category*	32				
N/A		18 (57%)			
B		8 (25%)			
C		6 (19%)			
Cerebral HIV/AIDS	32	3 (9%)			
Peak HIV VL log copies/mL	29	5.13 (4.65–5.68)			
Nadir CD4+ *Z* score	30	-0.83 (0.58)			
HIV viral load at eye exam	32				
Detectable		4 (13%)			
Undetectable		28 (88%)			
Age at cART initiation (y)	29	3.0 (1.2–5.6)			
Current cART use	32	31 (97%)			
Duration cART use (y)	29	9.1 (7.1–16.3)			

Numbers represent the mean and standard deviation or median and interquartile range.

Abbreviations: AIDS = acquired immunodeficiency syndrome; cART = combination antiretroviral therapy; CDC = Centers for Disease Controls and Prevention; HIV = human immunodeficiency virus; n = number of patients; OCT = optical coherence tomography; VL = viral load; y = years.

Statistical tests

^A^ = Student’s *t* test

^B^ = Fisher’s exact test

^C^ = Mann-Whitney *U* test.

* per eye.

ƚ Centers for Disease Control and Prevention (CDC) category: N not symptomatic; A mildly symptomatic; B moderately symptomatic; C severely symptomatic.

Numbers are mean and standard deviation or median and interquartile range.

Abbreviations: AIDS = acquired immunodeficiency syndrome; cART = combination antiretroviral therapy; CDC = Centers for Disease Control and Prevention; HIV = human immunodeficiency virus; n = number of patients; OCT = optical coherence tomography; VL = viral load; y = years.

Statistical tests

^A^ = Student’s *t* test

^B^ = Fisher’s exact test

^C^ = Mann-Whitney *U* test.

* Centers for Disease Control and Prevention (CDC) category: N not symptomatic; A mildly symptomatic; B moderately symptomatic; C severely symptomatic.

The median age at follow-up was 17.5 years (interquartile range [IQR]: 15.5–20.7) for the PHIV adolescents and 16.4 years (IQR 15.8–19.5) for controls. Of the PHIV adolescents, 7 (33%) were adopted, which was significantly different compared to the controls where no adolescents were adopted. A majority of participants in both groups was Black (PHIV 76%; controls 70%). The median age at HIV diagnosis was 1.7 years (IQR 0.8–4.2). At the first assessment two participants had a detectable HIV VL. At the second assessment four participants had a detectable HIV VL of which one participant had a detectable HIV VL at both visits. The median age at cART initiation was 2.5 years. At time of the second assessment 95% used cART with a median duration of 14.9 years. In the adolescents who did not participate in the follow-up study, we found significant lower fovea thickness in PHIV adolescents compared to controls, demonstrating selective drop-out (PHIV: 223μm, IQR 218–231, controls: 250μm, IQR 240–261, *p* < 0.001). We did not find selective dropout in other ophthalmologic outcomes or demographic parameters.

### Demographic characteristics of the cross-sectional substudy

Table 1B presents the demographic characteristics of the participants in the cross-sectional study. We included 32 PHIV children or adolescents and 34 controls in the cross-sectional study of which 21 PHIV children or adolescents and 19 controls also participated in the longitudinal substudy. One participant from the PHIV group was excluded because of a suspected ocular disease on OCT scan. The median age of the PHIV group was 15.4 years (IQR 11.2–19.2) and 15.3 years (IQR 10.9–17.6) of the controls. 17 (53%) of the cases were adopted which was not significantly different compared to the controls. The median age at HIV diagnosis was 2.1 years (IQR: 0.8–4.1). Four participants (13%) had a detectable HIV viral load during the eye exam.

### Longitudinal analyses

**Fig 3:**
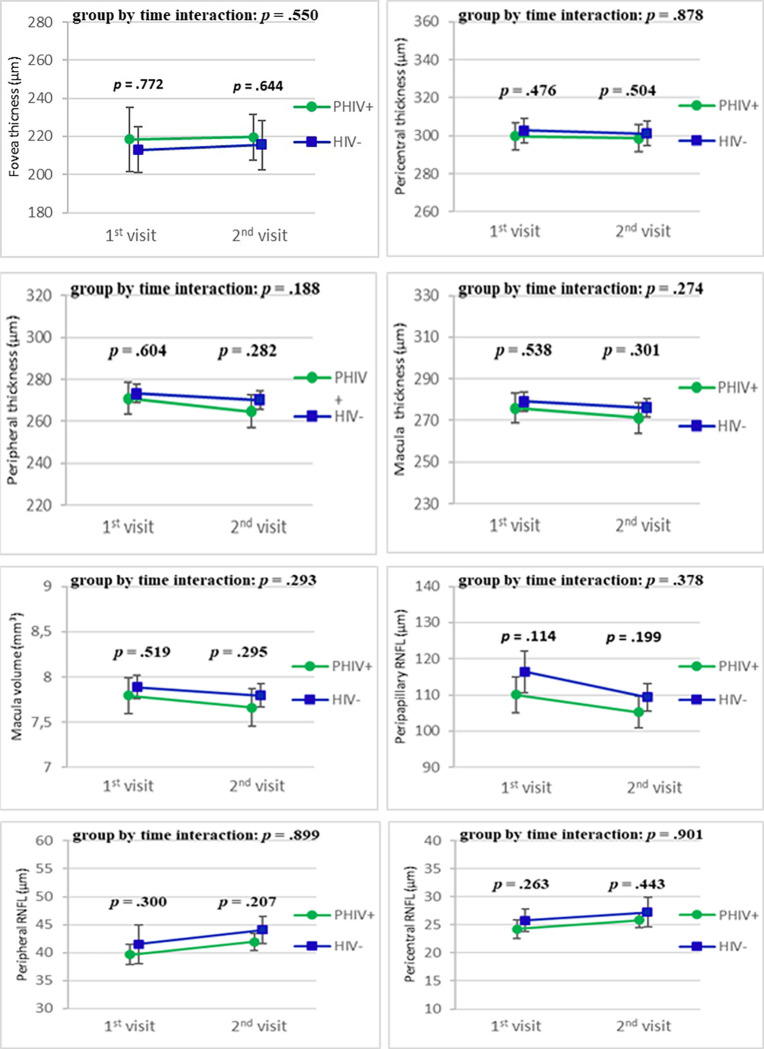
Longitudinal changes over time in retina thickness and volume in PHIV children and healthy controls. The figure shows changes between the first and follow-up OCT measurements in HIV-infected children (PHIV + in green) and healthy controls (HIV- in blue). The graphs show the means with the 95% confidence interval and p-values of the group-by-time interaction and at both visits using linear (mixed) regression model adjusted for sex and age; RNFL = retina nerve fibre layer.

Table 2A and 2B and Fig 3 shows the results of the difference in OCT outcomes in PHIV adolescents and healthy controls over time. There were no significant alterations in the following OCT outcomes over time: fovea thickness, pericentral thickness, peripheral thickness, RNFL thickness, pRNFL thickness and the macula volume. We found that changes of the pRNFL over time were associated with changes in FA and RD when taking the PHIV and healthy children and adolescents together. (S1 File).

**Table 2:** a. OCT Individual Retina Layer Thickness in PHIV Children and Healthy Controls, longitudinal substudy visit 1. b. OCT Individual Retina Layer Thickness in PHIV Children and Healthy Controls, longitudinal substudy visit 2.

**Visit 1**	**PHIV children, nr = 20**		**Healthy Controls, nr = 19**					
Macular layer	n	Mean(SD)/Median(IQR)	n	Mean(SD)/Median(IQR)	Coefficient	95%CI	*p*	ES
Fovea RT	20	218.5 (20.1)	19	213 (207.2–231)	-2.052	-16.328–12.225	.772	.060
Pericentral RT	20	299.7 (15,6)	19	302.7 (13.4)	-3.499	-13.345–6.348	.476	-.208
Peripheral RT	19	275.2 (258.8–283.8)	19	274 (9.0)	-2.283	-11.138–6.572	.604	-.242
Total RT	20	279.9 (264.9–288.5)	18	279.1 (273.4–284.4)	-2.619	-11.184–5.946	.538	-.250
Total volume	19	7.8 (7.5–8.2)	19	7.9 (7.7–8.0)	-0.077	-0.318–0.164	.519	-.259
RNFL								
Pericentral	16	25.4 (22.8–26.0)	18	25.2 (23.5–28.1)	-1.483	-4.140–1.173	.263	-.421
Peripheral	15	40 (38.1–42.5)	16	43.4 (39.8–44.8)	-2.060	-6.056–1.937	.300	-.354
pRNFL	17	110 (9.8)	18	114 (107–126.9)	-6.287	-14.165–1.591	.114	-.595
**Visit 2**	**PHIV children, nr = 20**		**Healthy Controls, nr = 19**					
Macular layer	n	Mean(SD)/Median(IQR)	n	Mean(SD)/Median(IQR)	Coefficient	95%CI	*p*	ES
Fovea RT	20	219.6 (21.4)	19	215.5 (204–237.2)	-3.300	-17.667–11.068	.664	-.125
Pericentral RT	20	298.7 (15.2)	19	301.2 (13.2)	-3.205	-12.850–6.440	.504	-.177
Peripheral RT	19	270.5 (251.3–277.6)	19	270.1 (8.8)	-4.921	-14.068–4.227	.282	-.405
Total RT	20	275.1 (258–283.4)	18	275.9 (9.4)	-4.607	-13.513–4.300	.301	-.368
Total volume	19	7.8 (7.3–8.0)	19	7.8 (0.3)	-0.131	-0.381–0.119	.295	-.372
RNFL								
Pericentral	16	26.3 (24.3–28)	18	25.7 (24.1–28.8)	-1.155	-4.192–1.882	.443	-.312
Peripheral	15	42.8 (41.5–43.4)	16	42.6 (41.8–45.2)	-1.811	-.4.688–1.066	.207	-.555
pRNFL	17	105.3 (8.5)	18	109.3 (7.6)	-3.792	-9.681–2.098	.199	-.497

Mean and median expressed in μm.

*p* value was measured using multivariable linear regression adjusting for age and sex. Effect size thresholds: (-)d; 0.01 = very small, 0.2 = small, 0.5 = medium, 0.8 = large.

Abbreviations: CI = 95% confidence interval; IQR = interquartile range; n = number of eyes; nr = number of patients; pRNFL = peripapillary retinal nerve fibre layer; RNFL = retinal nerve fibre layer; RT = retina thickness; SD = standard deviation.

### Structure of the retina in the cross-sectional substudy

We found comparable thickness of individual retinal layer thickness of the macula and the pRNFL in the PHIV children and controls (Table 3). Three scans of the macula and one of the pRNFL were excluded because of low quality. In eleven scans measurements of some retinal layers were excluded due to technical errors. We found no significant differences in the thickness and volume of the retina.

**Table 3:** OCT individual retina layer thickness in PHIV children and healthy controls, cross-sectional substudy.

	**PHIV children, nr = 32**		**Healthy Controls, nr = 34**					
Macular layer	n	Mean(SD)/Median(IQR)	n	Mean(SD)/Median(IQR)	Coefficient	95%CI	*p*	ES
Fovea RT	62	254.5 (20.4)	66	259.7 (19.2)	-5.566	-15.477–4.344	.266	-.261
Pericentral RT	62	336.1 (16.9)	65	338.7 (13.7)	-3.662	-11.037–3.712	.325	-.172
Peripheral RT	61	304.2 (291.8–316.3)	59	307.2 (296.5–311.4)	-1.110	-8.477–6.258	.764	-.047
Total volume	61	8.8 (8.4–9.1)	60	8.8 (8.6–8.9)	-0.048	-0.246–0.150	.627	-.083
Volume fovea	62	0.2 (0.2–0.2)	66	0.2 (0.2–0.2)	-0.005	-0.013–0.003	.235	-.270
Volume pericentral	62	2.1 (0.1)	65	2.1 (0.1)	-0.024	-0.070–0.023	.311	-.183
Volume peripheral	61	6.4 (6.2–6.7)	59	6.5 (6.3–6.6)	-0.023	-0.179–0.134	.774	-.043
RNFL								
Fovea	62	10.5 (2.5)	66	11.3 (2.3)	-0.735	-15.477–4.344	.222	-.304
Pericentral	62	20.1 (1.4)	66	20.6 (1.5)	-0.570	-1.269–0.129	.108	-.349
Peripheral	61	33.2 (3.5)	60	34.2 (3.8)	-1.101	-2.889–0.688	.223	-.260
GCL								
Fovea	62	11.8 (9.4–14.5)	66	12.5 (10.6–16.9)	-0.087ƚ	-0.236–0.062	.246	-.298
Pericentral	62	52.1 (50.1–54.0)	66	53.1 (50.8–55.8)	-1.779	-3.965–0.408	.109	-.360
Peripheral	61	38.9 (35.8–41.0)	61	39.0 (36.9–40.1)	0.150	-1.707–2.006	.873	.064
IPL								
Fovea	62	18.0 (16.0–18.7)	66	18.3 (17.5–21.0)	-0.032ƚ	-0.118–0.054	.459	-.183
Pericentral	62	42.4 (40.0–43.8)	66	42.5 (40.8–44.0)	-0.492	-2.022–1.039	.523	-.114
Peripheral	61	31.1 (29.3–33.5)	61	31.3 (30.1–32.9)	0.159	-1.275–1.592	.826	.072
INL								
Fovea	62	14.0 (11.0–17.3)	66	15.5 (12.5–19.0)	-1.605	-3.822–0.612	.153	-.345
Pericentral	62	39.6 (35.5)	66	39.7 (2.9)	-0.215	-1.794–1.364	.786	-.029
Peripheral	61	35.1 (33.3–36.3)	61	34.8 (33.8–36.4)	0.042	-1.314–1.398	.951	.026
OPL								
Fovea	62	20.5 (18.0–24.0)	66	20.5 (18.8–24.0)	-0.008ƚ	-0.114–0.097	.874	-.061
Pericentral	62	31.4 (28.5–33.2)	66	30.2 (28.9–31.5)	0.905	-0.558–2.367	.221	.275
Peripheral	61	26.4 (1.6)	61	25.8 (1.3)	0.580	-0.173–1.332	.129	.389

	**PHIV children, nr = 32**		**Healthy Controls, nr = 34**					
	n	Mean(SD)/Median(IQR)	n	Mean(SD)/Median(IQR)	Coefficient	95%CI	*p*	ES
ONL								
Fovea	62	91.0 (82.0–97.3)	66	92.3 (81.4–96.5)	-1.862	-6.556–2.831	.431	-.124
Pericentral	62	71.4 (7.8)	66	72.5 (7.1)	-1.496	-5.136–2.145	.415	-.140
Peripheral	61	60.7 (6.7)	61	61.1 (5.6)	-0.464	-3.524–2.598	.763	-.069
RPE								
Fovea	62	16.5 (15.9–17.6)	66	16.5 (15.5–17.5)	0.006ƚ	-0.038–0.051	.779	.122
Pericentral	62	14.2 (1.8)	65	13.7 (1.3)	0.448	-0.220–1.116	.185	.401
Peripheral	61	12.4 (11.6–13.0)	60	11.8 (11.4–12.6)	0.017ƚ	-0.040–0.074	.550	.111
IRL								
Fovea	62	165.8 (153.6–175.4)	66	166.0 (154.1–186.4)	-6.090	-15.864–3.685	.218	-.299
Pericentral	62	257.1 (246.0–266.6)	66	259.5 (252.4–262.5)	-3.780	-10.696–3.136	.279	-.206
Peripheral	61	228.2 (213.0–239.0)	61	227.5 (219.8–233.2)	-0.955	-8.436–6.527	.779	-.041
PR								
Fovea	62	90.1 (3.8)	66	89.4 (4.0)	0.584	-1.351–2.518	.549	.190
Pericentral	62	80.3 (78.7–82.3)	65	79.9 (78.6–81.4)	0.067	-1.055–1.189	.906	.106
Peripheral	61	77.2 (76.3–78.4)	60	77.4 (75.8–78.3)	-0.005ƚ	-0.021–0.010	.513	-.146
pRNFL								
Total	64	103.3 (13.8)	67	104.8 (10.1)	-1.271	-7.135–4.594	.667	.0122
Superior	64	131.0 (23.5)	67	133.5 (17.7)	-2.517	-12.832–7.797	.627	-.118
Inferior	64	132.7 (123.1–146.2)	67	139.3 (128.8–146.1)	-0.826	-9.467–7.815	.849	-.083
Temporal	64	69.8 (63.9–77.7)	67	75.4 (67.6–82.6)	-3.826	-9.319–1.666	.169	-.375
Nasal	64	76.8 (66.3–85.3)	67	72.3 (62.9–81.6)	2.707	-4.468–9.881	.454	.190

Mean and median expressed in μm.

*p* value was measured using multivariable linear regression adjusting for age and sex. When data was not normally distributed we used logarithmic transformation to approach a normal distribution. Effect size thresholds: (-)d; 0.01 = very small, 0.2 = small, 0.5 = medium, 0.8 = large.

Abbreviations: CI = 95% confidence interval; GCL = ganglion cell layer; INL = inner nuclear layer; IPL = inner plexiform layer; IRL = inner retinal layer; IQR = interquartile range; n = number of eyes; nr = number of patients; ONL = outer nuclear layer; OPL = outer plexiform layer; PR = photoreceptors; pRNFL = peripapillary retinal nerve fibre layer; RNFL = retinal nerve fibre layer; RPE = retinal pigment epithelium; RT = retina thickness; SD = standard deviation.

ƚ = log.

### Associations with OCT parameters in the cross-sectional substudy

In the S1 File the associations between the OCT parameters and the HIV- and cART-related characteristics and MRI parameters (including cases and controls) are shown. We found that a thinner peripheral layer and the pericentral GCL were significantly associated with a lower FA. We did not find significant associations between the thickness of the retina and the HIV- and cART-related characteristics.

## Discussion

In this study we found a similar development in retina structure in PHIV adolescents and controls with a mean follow-up of 4.6 years. Cross-sectionally, we also found a comparable RT between PHIV children and controls. In our association analyses we found that changes in the pRNFL was associated with changes of the FA and RD. Cross-sectionally, we found that a thinner peripheral retina was associated with a lower FA.

While using similar methodology, we did not find differences in RT alterations between PHIV children or adolescents and controls over time, suggesting a normal development of the retina. A longitudinal study in healthy ageing adolescents and adults reported no changes in fovea thickness [24]. Due to selective dropout, the development of the fovea thickness remains unanswered in PHIV children and adolescents within this study. Previous cross-sectional studies comparing PHIV children to controls reported contrasting yet subtle differences in RT, including a thinner fovea, a thinner pRNFL and a thicker fovea compared to controls [3–5]. Possible explanations for not finding these differences could be the different study population and their characteristics. Our participants had a lower peak VL (5.13 vs. 5.54 log copies/ml) and previously, a higher VL was associated with a thinner fovea (-10.7 μm per log copy/mL, p value = 0.016) [5]. In the current study, the participants had a shorter duration of cART use. Possible cART-associated retinopathy was previously described for didanosine and stavudine [25], however we do not have complete historical data to assess this association due to the majority of participants being adopted. We hypothesized that the use of different OCT equipment could have also contributed to differences with other studies. In previous studies a time-domain OCT (TD-OCT) was used to measure the RT [3,4]. However, a study evaluating the quality of time-domain and SD-OCT demonstrated less accurate outcomes in the TD-OCT [26]. Studies in HIV-infected adults also exhibited contrasting results in macula thickness; they hypothesized that hypoxia or oedema caused a thinner or thicker macula, respectively [27].

Over time, we found an association between changes in the pRNFL and FA and an inverse association between changes in the pRNFL and RD indicating a relation between changes in the pRNFL over time and changes in WM integrity. This finding contrasts our baseline study, in which such association was not detected [10]. We hypothesized this difference could be explained by relatively small differences in WM integrity between groups cross-sectionally, however longitudinally the WM integrity differences persist and could lead to detecting subtle association. The pRNFL consists of axons of the ganglion cells forming the optic nerve, which is therefore closely related to the WM structure [28]. These changes in WM integrity and pRNFL could tell us more about disease-related pathophysiology, or could be part of physiological WM neuroplasticity [29]. Our results demonstrate a potential relation between the retina and the brain over time, which suggests that OCT should be performed additionally when brain changes or pathology are detected on MRI.

Cross-sectionally, we did not find associations between RT and HIV-related variables. Although a type II error cannot be ruled out, the lack of associations paints an optimistic picture that living with perinatal HIV or using cART may not necessarily be associated with RT differences. It also adds to previous scarce and inconclusive evidence [3–5]. Nonetheless, the evaluation of the retina should not be completely omitted, however a lower frequency of analysis would suffice. We found a thinner peripheral area to the retina to be associated with a lower FA, indicating a lower WM integrity, the precise clinical interpretation is hampered as FA is highly sensitive but not very specific [30]. Larger studies are needed to confirm these results and to elucidate the potentially shared relation between the retina and potential brain pathology.

Our results should be interpreted with caution; due to multiple testing there is possible risk of Type I errors. As our analyses are explorative, we did not use the Bonferroni correction [31]. While including about 20% of Dutch pediatric HIV cases, the study however has a relatively small sample size, which resulted in the study to be considered underpowered.

This study has multiple strengths, including a well-matched healthy control group. It is the first study to longitudinally explore retinal development in PHIV children and adolescents. In addition, we assessed all individual layers of the retina and evaluated the four different quadrants of the pRNFL for the cross-sectional study using high quality OCT devices. We also report some important limitations of this study. The small number of participants of this study might have impeded the possibility of detecting subtle differences between the two groups. Moreover the small sample size also reduced the generalizability of this study and prevented the use of larger multivariable models [32]. Furthermore we were unable to assess the individual layers of the retina in the longitudinal substudy. The cross-sectional study design does not allow us to causally link established identified factors to RT [33]. Finally we do not have historical data from children arriving in the Netherlands at older age.

## Conclusions

This study suggests normal retinal structure development in ageing PHIV children and adolescents. We found significant associations between the development of the retina and the brain, suggesting a shared physiologic development irrespective of HIV-infection once under control with cART.

## Supporting information

S1 File (DOCX)
