# Supplementary material for: Development of retinal structure in perinatally HIV-infected children and adolescents: A longitudinal and cross-sectional assessment
Source: PLoS One. 2023 Mar 2;18(3):e0282284. doi: 10.1371/journal.pone.0282284 (PMC9980730; doi:10.1371/journal.pone.0282284)
Supplement: S1 File — (DOCX) [file pone.0282284.s001.docx]

**Table S1. Associations between longitudinal changes over time in retina thickness and DTI parameters in PHIV children and healthy controls**

|  | n | Fractional Anisotropy | | | Mean Diffusivity | | | Radial Diffusivity | | | Axial Diffusivity | | |
| --- | --- | --- | --- | --- | --- | --- | --- | --- | --- | --- | --- | --- | --- |
| Retina |  | Coefficient | 95%CI | *p* | Coefficient | 95%CI | *p* | Coefficient | 95%CI | *p* | Coefficient | 95%CI | *p* |
| Fovea | 39 | 0.014 | -0.000 - 0.027 | .064 | -0.162 | -0.368 - 0.045 | .170 | -0.197 | -0.411 - 0.019 | .106 | -0.109 | -0.404 - 0.185 | .499 |
| Pericentral | 39 | 0.013 | -0.008 - 0.033 | .259 | 0.001 | -0.315 - 0.328 | .995 | -0.129 | -0.472 - 0.225 | .478 | 0.161 | -0.252 - 0.582 | .474 |
| Peripheral | 39 | 0.005 | -0.019 - 0.030 | .675 | 0.041 | -0.312 - 0.398 | .836 | -0.005 | -0.375 - 0.369 | .980 | 0.101 | -0.388 - 0.593 | .706 |
| Thickness | 39 | 0.008 | -0.016 - 0.033 | .523 | 0.031 | -0.326 - 0.395 | .876 | -0.046 | -0.424 - 0.340 | .826 | 0.128 | -0.363 - 0.624 | .635 |
| Volume | 39 | 0.312 | -0.570 - 1.191 | .509 | 1.056 | -11.650 - 13.992 | .882 | -1.674 | -15.131 - 12.038 | .822 | 4.548 | -12.941 - 22.198 | .636 |
| Pericentral RNFL | 39 | -0.006 | -0.049 - 0.035 | .775 | -0.487 | -1.477 - 0.608 | .364 | -0.332 | -1.215 - 0.635 | .483 | -0.860 | -2.247 - 0.638 | .252 |
| Peripheral RNFL | 39 | -0.001 | -0.035 - 0.032 | .969 | -0.201 | -1.063 - 0.804 | .666 | -0.152 | -0.886 - 0.719 | .699 | -0.420 | -1.635 - 0.945 | .521 |
| Peripapillary RNFL | 39 | 0.030 | 0.006 - 0.054 | **.022*** | -0.468 | -0.955 - 0.031 | .085 | -0.568 | -1.025 to -0.105 | **.025*** | -0.284 | -0.965 - 0.407 | .445 |

Coefficients represents the change in DTI parameters (FA x 10^2^, MD/RD/AD x 10^6^) per micron increase retina thickness over time;

* p < 0.05; p value was measured using mixed models adjusting for status, sex and age

Abbreviations: CI = confidence interval; n = number of participants including PHIV children or adolescents and controls; RNFL = retina nerve fibre layer

**Table S2a. Associations between RT and HIV- and cART-related characteristics, cross-sectional substudy**

|  |  | **Peak HIV viral load** | | | **Nadir Z-score CD4+** | | | **Age cART initiation** | | | **Duration cART use** | | |
| --- | --- | --- | --- | --- | --- | --- | --- | --- | --- | --- | --- | --- | --- |
|  | n | Coefficient | 95%CI | *p* | Coefficient | 95%CI | *p* | Coefficient | 95%CI | *p* | Coefficient | 95%CI | *p* |
| Fovea layers |  | |  |  |  |  |  |  |  |  |  |  |  |
| Total RT | 29 | -0.466 | -1.687 - 0.756 | .440 | 5.157 | -9.165 - 19.479 | .466 | -0.246 | -2.580 - 2.089 | .830 | 0.237 | -2.097 - 2.570 | .836 |
| RNFL | 29 | -0.027 | -0.178 - 0.124 | .719 | 0.392 | 1.364 - 2.148 | .650 | 0.066 | -0.217 - 0.350 | .635 | -0.067 | -0.350 - 0.216 | .630 |
| GCL | 29 | -0.045 | -0.279 - 0.189 | .692 | -0.065 | -2.790 - 2.660 | .961 | 0.175 | -0.259 - 0.609 | .414 | -0.177 | -0.611 - 0.257 | .409 |
| Pericentral layers |  | |  |  |  |  |  |  |  |  |  |  |  |
| Total RT | 29 | -0.406 | -1.330 - 0.518 | .374 | 4.654 | -6.402 - 15.709 | .395 | 0.154 | 1.620 - 1.928 | .860 | -0.160 | -1.933 - 1.613 | .854 |
| RNFL | 29 | -0.041 | -0.121 - 0.038 | .293 | 0.435 | -0.517 - 1.387 | .356 | 0.025 | -0.131 - 0.181 | .745 | -0.025 | -0.181 - 0.131 | .744 |
| GCL | 29 | 0.025 | -0.239 - 0.289 | .846 | 0.835 | -2.245 - 3.915 | .582 | 0.358 | -0.120 - 0.836 | .135 | -0.360 | -0.837 - 0.117 | .133 |
| Peripheral layers |  | |  |  |  |  |  |  |  |  |  |  |  |
| Total RT | 29 | 0.021 | -0.937 - 0.980 | .964 | 5.200 | -5.902 - 16.301 | .345 | 0.897 | -0.769 - 2.564 | .278 | -0.903 | -2.567 - 0.762 | .275 |
| RNFL | 29 | -0.069 | -0.252 - 0.115 | .450 | 0.940 | -1.271 - 3.150 | .390 | 0.271 | -0.074 - 0.616 | .118 | -0.272 | -0.617 - 0.073 | .117 |
| GCL | 29 | 0.118 | -0.124 - 0.361 | .324 | 1.050 | -1.826 - 3.926 | .460 | 0.396 | -0.028 - 0.820 | .066 | -0.398 | -0.821 - 0.026 | .065 |
| Total pRNFL | 29 | 0.400 | -0.355 - 1.154 | .286 | 4.129 | -5.255 - 13.512 | .374 | 1.200 | -0.173 - 2.573 | .084 | 1.207 | -2.578 - 0.163 | .082 |

HIV- and cART-related characteristics: coefficients represent changes in thickness (µm) per increase peak HIV VL (copies/mL), CD4+ T-cell count (*Z* score), age cART initiation (years) and duration cART treatment (years); Association analyses using multivariable linear regression adjusting for age, sex and HIV status; * p < 0.05

Abbreviations: cART = combination antiretroviral therapy; GCL = ganglion cell layer; HIV = human immunodeficiency virus; RNFL = retinal nerve fibre layer RT = retina thickness; pRNFL = peripapillary retina nerve fibre layer

**Table S2b. Associations between RT and DTI parameters, cross-sectional substudy in PHIV children and healthy controls**

|  |  | Fractional Anisotropy | | | Mean Diffusivity | | | Radial Diffusivity | | | Axial Diffusivity | | |
| --- | --- | --- | --- | --- | --- | --- | --- | --- | --- | --- | --- | --- | --- |
|  | n | Coefficient | CI 95% | *p* | Coefficient | CI 95% | *p* | Coefficient | CI 95% | *p* | Coefficient | CI 95% | *p* |
| Fovea layers |  |  |  |  |  |  |  |  |  |  |  |  |  |
| Total RT | 52 | 0.012 | -0.005 - 0.029 | .152 | -0.125 | -0.385 - 0.135 | .339 | -0.163 | -0.446 - 0.121 | .254 | -0.050 | -0.347 - 0.247 | .738 |
| RNFL | 52 | 0.090 | -0.053 - 0.233 | .211 | -1.031 | -3.201 - 1.138 | .344 | -1.210 | -3.585 - 1.164 | .310 | -0.675 | -3.149 - 1.799 | .586 |
| GCL | 52 | 0.066 | -0.029 - 0.154 | .142 | -1.011 | -2.346 - 0.324 | .134 | -1.129 | -2.610 - 0.352 | .132 | -0.829 | -2.360 - 0.701 | .281 |
| Pericentral layers |  |  |  |  |  |  |  |  |  |  |  |  |  |
| Total RT | 52 | 0.019 | -0.003 - 0.042 | .095 | -0.004 | -0.350 - 0.358 | .983 | -0.126 | -0.511 - 0.258 | .511 | 0.259 | -0.134 - 0.653 | .192 |
| RNFL | 52 | 0.235 | -0.012 - 0.483 | .062 | -2.644 | -6.449 - 1.162 | .169 | -3.607 | -7.717 - 0.502 | .084 | -0.831 | -5.224 - 3.563 | .705 |
| GCL | 52 | 0.094 | 0.019 - 0.168 | **.015*** | -0.772 | -1.950 - 0.406 | .194 | -1.155 | -2.419 - 0.110 | .073 | -0.008 | -1.367 - 1.351 | .990 |
| Peripheral layers |  |  |  |  |  |  |  |  |  |  |  |  |  |
| Total RT | 52 | 0.025 | 0.003 - 0.047 | **.026*** | -0.205 | -0.552 - 0.142 | .241 | -0.334 | -0.707 - 0.039 | .078 | 0.034 | -0.370 - 0.439 | .865 |
| RNFL | 52 | 0.104 | 0.006 - 0.203 | **.038*** | -1.257 | -2.763 - 0.250 | .099 | -1.621 | -2.886 - 0.233 | .051 | -0.601 | -2.371 - 1.170 | .498 |
| GCL | 52 | 0.086 | -0.002 - 0.174 | .056 | -1.040 | -2.387 - 0.308 | .127 | -1.363 | -2.808 - 0.081 | .064 | -0.397 | -1.977 - 1.184 | .616 |
| pRNFL | 52 | 0.026 | -0.002 - 0.054 | .067 | -0.096 | -0.531 - 0.338 | .658 | -0.272 | -0.751 - 0.208 | .261 | 0.215 | -0.275 - 0.704 | .383 |

DTI parameters: coefficients represent the change in DTI parameters (FA x 10², MD/RD/AD x 10^6^) per micron increase RT;

Association analyses using multivariable linear regression adjusting for age, sex and HIV status; * p < 0.05

Abbreviations: DTI = diffusion tensor imaging; n = number of participants including PHIV children or adolescents and controls; GCL = ganglion cell layer; pRNFL = peripapillary retinal nerve fibre layer; RT = retina thickness; RNFL = retinal nerve fibre layer;

Post-hoc, it was decided to present the following tables separately in the Supplementary Materials. It should be noted that the number of association analyses is rather large. Hence this could lead to Type I errors. However, as these analyses have been performed as part of the original methods, we decided to include these analyses in the following section.

A notable result in Table S3 shows that over time changes in RT were associated with changes in GM volume and changes in the pRNFL were inversely associated with changes in GM volume. In children the GM volume increases before adolescence and decreases when growing into adulthood which might explain the inverse association between the pRNFL and GM volume [1]. A study performed in healthy adults reported positive associations between GM volume and the pRNFL, which contrasts our findings, probably due to the difference between the development of the brain in children and adults [2]. In a recent study an association was found between the RT and GM volume in adults with multiple sclerosis, which is in line with our findings and might reflect the same neuroinflammatory pathogenesis in the retina and brain [3].

**Table S3. Associations between longitudinal changes over time in retina thickness and brain volume in PHIV children and healthy controls**

|  | n | GM Volume | | | WM Volume | | | WMH volume log | | |
| --- | --- | --- | --- | --- | --- | --- | --- | --- | --- | --- |
| Retina |  | Coefficient | 95%CI | *p* | Coefficient | 95%CI | *p* | Coefficient | 95%CI | *p* |
| Fovea | 39 | -0.029 | -0.088 - 0.033 | .360 | 0.014 | -0.022 - 0.050 | .459 | 0.003 | -0.002 - 0.009 | .199 |
| Pericentral | 39 | 0.119 | 0.027 - 0.212 | **.017*** | 0.010 | -0.049 - 0.071 | .749 | -0.003 | -0.012 - 0.006 | .458 |
| Peripheral | 39 | 0.132 | 0.031 - 0.234 | **.016*** | -0.013 | -0.077 - 0.053 | .691 | 0.003 | -0.006 - 0.013 | .490 |
| Thickness | 39 | 0.141 | 0.035 - 0.247 | **.014*** | -0.002 | -0.069 - 0.068 | .959 | 0.002 | -0.007 - 0.012 | .684 |
| Volume | 39 | 5.010 | 1.260 - 8.783 | **.014*** | -0.194 | -2.591 - 2.295 | .878 | 0.070 | -0.267 - 0.423 | .693 |
| Pericentral RNFL | 39 | 0.186 | 0.050 - 0.322 | **.012*** | 0.013 | -0.064 - 0.093 | .741 | -0.008 | -0.026 - 0.008 | .343 |
| Peripheral RNFL | 39 | 0.071 | -0.039 - 0.180 | .217 | -0.010 | -0.067 - 0.048 | .729 | -0.015 | -0.030 - 0.000 | .070 |
| Peripapillary RNFL | 39 | -0.113 | -0.204 to -0.018 | **.023*** | -0.011 | -0.063 - 0.041 | .683 | -0.000 | -0.007 - 0.007 | .954 |

Coefficients represents the change in volume (GM/WM volume x 10^2^ and WMH in cm^3^) per micron increase retina thickness over time

* p < 0.05; p value was measured using mixed models adjusting for status, sex and age.

Abbreviations: CI = confidence interval; GM = grey matter; n = number of participants including PHIV children or adolescents and controls; RNFL = retina nerve fibre layer; WM = white matter; WMH = white matter hyperintensities

**Table S4. Associations between RT and Cerebral Volume, cross-sectional substudy in PHIV children and healthy controls**

|  |  | **GM Volume** | | | **WM Volume** | | | **WMH volume, log** | | |
| --- | --- | --- | --- | --- | --- | --- | --- | --- | --- | --- |
|  | n | Coefficient | CI 95% | *p* | Coefficient | 95%CI | *p* | Coefficient | CI 95% | *p* |
| Fovea layers |  |  |  |  |  |  |  |  |  |  |
| Total RT | 58 | 0.018 | -0.054 - 0.090 | .623 | 0.010 | -0.052 - 0.072 | .741 | 0.006 | -0.005 - 0.016 | .281 |
| RNFL | 58 | 0.325 | -0.266 - 0.915 | .275 | 0.240 | -0.267 - 0.748 | .346 | 0.004 | -0.086 - 0.095 | .924 |
| GCL | 58 | 0.117 | -0.245 - 0.480 | .519 | 0.102 | -0.209 - 0.413 | .513 | 0.008 | -0.047 - 0.063 | .769 |
| Pericentral layers |  |  |  |  |  |  |  |  |  |  |
| Total RT | 58 | 0.049 | -0.048 - 0.146 | .313 | 0.022 | -0.062 - 0.105 | .605 | 0.001 | -0.012 - 0.014 | .867 |
| RNFL | 58 | -0.090 | -1.129 - 0.949 | .863 | 0.452 | -0.430 - 1.334 | .309 | -0.004 | -0.147 - 0.138 | .950 |
| GCL | 58 | 0.193 | -0.139 - 0.525 | .249 | 0.221 | -0.061 - 0.502 | .122 | -0.039 | -0.056 - 0.031 | .560 |
| Peripheral layers |  |  |  |  |  |  |  |  |  |  |
| Total RT | 58 | 0.031 | -0.067 - 0.130 | .525 | 0.031 | -0.052 - 0.115 | .457 | 0.003 | -0.009 - 0.015 | .613 |
| RNFL | 58 | -0.063 | -0.483 - 0.356 | .764 | 0.189 | -0.164 - 0.542 | .288 | 0.005 | -0.048 - 0.059 | .841 |
| GCL | 58 | 0.142 | -0.258 - 0.541 | .480 | 0.176 | -0.161 - 0.514 | .300 | 0.000 | -0.049 - 0.050 | .984 |
| pRNFL | 58 | 0.102 | -0.023 - 0.226 | .108 | 0.117 | 0.012 - 0.231 | **.030*** | 0.009 | -0.006 - 0.025 | .227 |

Cerebral volumes: coefficients represent changes in volume (GM/WM x 10^2^, WMH x 10 in cm^3^ ) per micron increase in RT

Association analyses using multivariable linear regression adjusting for age, sex and HIV status; * p < 0.05

Abbreviations: GCL = ganglion cell layer; GM = grey matter; pRNFL = peripapillary retinal nerve fibre layer; RNFL = retinal nerve fibre layer; RT = retina thickness; WM = white matter; WMH = white matter hyperintensities

**References**

1. Taki Y, Kawashima R. Brain development in childhood. Open Neuroimag J. 2012;6:103-10.

2. Shi Z, Zheng H, Hu J, Jiang L, Cao X, Chen Y, et al. Retinal Nerve Fiber Layer Thinning Is Associated With Brain Atrophy: A Longitudinal Study in Nondemented Older Adults. Front Aging Neurosci. 2019;11:69.

3. Longitudinal analysis of retinal and brain atrophy in multiple sclerosis. Investigative opthalmology & visual science2021.
